# Ubiquitin-mediated regulation of APE2 protein abundance

**DOI:** 10.1016/j.jbc.2024.107337

**Published:** 2024-05-04

**Authors:** Anne McMahon, Jianjun Zhao, Shan Yan

**Affiliations:** 1 Department of Biological Sciences, University of North Carolina at Charlotte, Charlotte, North Carolina, USA; 2 Department of Cancer Biology, Lerner Research Institute, Cleveland Clinic, Cleveland, Ohio, USA; 3 School of Data Science, University of North Carolina at Charlotte, Charlotte, North Carolina, USA; 4 Center for Biomedical Engineering and Science, University of North Carolina at Charlotte, Charlotte, North Carolina, USA

**Keywords:** APE2, DNA damage response, MKRN3, proteostasis, post-translational modification (PTM), ubiquitin

## Abstract

APE2 plays important roles in the maintenance of genomic and epigenomic stability including DNA repair and DNA damage response. Accumulating evidence has suggested that APE2 is upregulated in multiple cancers at the protein and mRNA levels and that APE2 upregulation is correlative with higher and lower overall survival of cancer patients depending on tumor type. However, it remains unknown how APE2 protein abundance is maintained and regulated in cells. Here, we provide the first evidence of APE2 regulation *via* the posttranslational modification ubiquitin. APE2 is poly-ubiquitinated *via* K48-linked chains and degraded *via* the ubiquitin-proteasome system where K371 is the key residue within APE2 responsible for its ubiquitination and degradation. We further characterize MKRN3 as the E3 ubiquitin ligase for APE2 ubiquitination in cells and *in vitro*. In summary, this study offers the first definition of the APE2 proteostasis network and lays the foundation for future studies pertaining to the posttranslational modification regulation and functions of APE2 in genome integrity and cancer etiology/treatment.

AP endonuclease 2 (APE2) is an emerging protein of interest due to its wide range of proposed roles in DNA repair and DNA damage response (DDR) signaling as well as its recently defined synthetic lethality phenotype with BRCA1^−/−^ and BRCA2^−/−^ (1, 2, 3, 4, 5). While APE2 is now known to be important for cellular processes such as ATR-Chk1 DDR signaling (6, 7, 8, 9, 10), complex 3’ termini resolution (1, 11, 12, 13), microhomology-mediated end joining (2), class switch recombination (14, 15), and somatic hypermutation (16), no studies to date provide information on APE2’s steady state concentration (4, 5, 17). In addition, APE2’s mRNA abundance has been found to be significantly upregulated in multiple cancers such as breast, uterine, kidney, lung, and liver compared with nonmalignant tissues (18, 19). Evidence at both the protein and mRNA levels shows APE2 upregulation in multiple myeloma cells compared with normal cells (20). Upregulation of APE2 mRNA is correlative with worse overall survival in breast, liver, and kidney cancer but better overall survival in lung, bladder, ovarian, and cervical cancer (4). It remains unknown if APE2 is a driver or passenger of cancer development. Of additional clinical relevance, chemotherapy treatment with the drug cisplatin treatment induces upregulation of APE2 protein abundance through a yet to be defined mechanism, while APE2 overexpression leads to acute kidney injury (AKI) due to mitochondrial disfunction and thereby limits the effective dose of cisplatin in clinical settings (21).

Accumulating evidence in bioinformatics and large-scale “omics” analyses has suggested that human APE2 is modified *via* posttranslational modifications (PTMs) including ubiquitination and phosphorylation (22, 23, 24, 25, 26, 27, 28). Proteolytic turnover of proteins is cellularly essential for the regulation of multiple processes including cell cycle, apoptosis, and the DDR signaling (29, 30). It is estimated that 80 to 90% of proteins are degraded by the ubiquitin-proteasome system (UPS) (31). Briefly, the UPS system involves three critical proteins (E1 ubiquitin-activating enzyme, E2 ubiquitin-conjugating enzyme, and E3 ubiquitin ligase) that ultimately catalyze covalent binding of the carboxy terminus of Ubiquitin (Ub) to a lysine residue on the substrate protein. Ub can be added in multisubunit chains, often referred to as poly-ubiquitin chains. Poly-ubiquitin chains are associated with protein degradation, the best defined of these chains being lysine 48 linked (K48) poly-ubiquitin chains, which are strongly associated with recruitment to and subsequent degradation of K48-tagged proteins by the proteolytic activity of the proteasome (32). Although it is hypothesized that APE2 protein is ubiquitinated (17, 33), there is no experimental evidence of APE2 ubiquitination and degradation mechanisms to date.

Here, we provide the evidence of APE2’s ubiquitination and regulation, specifically focusing on protein abundance regulation by the UPS. We identified the critical residue for APE2 ubiquitination and degradation and characterized the E3 ligase enzyme responsible for APE2 ubiquitination and associated abundance in cells and *in vitro*. Our work is the first experimental evidence of APE2 ubiquitination and degradation, providing new insights into APE2 proteostasis and PTM-mediated regulation.

## Results

### APE2 is ubiquitinated and degraded by the UPS

**Figure 1:**
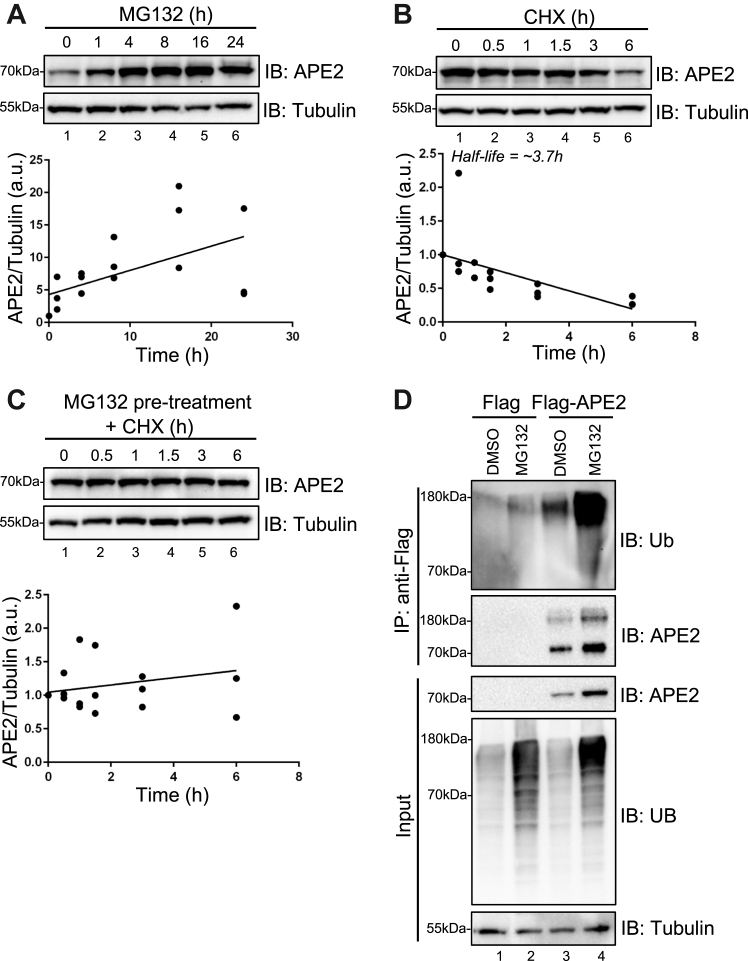
**APE2 is ubiquitinated and degraded by the ubiquitin-proteasomesystem.***A*, APE2 protein was immunoblotted, and APE2 protein levels were quantified (normalized to tubulin) from three biological replicates over a time course treatment of proteasome inhibitor MG132 in U2OS cells. Equation of line: Y = 0.3724∗X + 4.308, where Goodness of fit R square = 0.3029. *B*, APE2 protein was immunoblotted, and protein levels were quantified from three biological replicates over a time course treatment of translation inhibitor cycloheximide (CHX) in U2OS cells. Equation of line: Y = −0.1343∗X + 1.001, where Goodness of fit R square = 0.3969. *C*, APE2 protein was immunoblotted, and protein levels were quantified from three biological replicates over a time course treatment of translation inhibitor CHX with a 24h MG132 pretreatment in U2OS cells. Equation of line: Y = 0.05378∗X + 1.046 where Goodness of fit R square = 0.06829. *D*, Flag-APE2 or Flag expression plasmid was transfected to U2OS cells prior to MG132 treatment and Flag-IP. Immunoblotting (IB) analysis of proteins of interest from IP or Input samples as indicated. APE2, AP endonuclease 2.

To study the regulation of APE2 abundance, endogenous APE2 protein levels were analyzed *via* immunoblotting to detect the relative protein expression in human osteosarcoma U2OS cells under multiple experimental conditions (Fig. 1, *A–C*). Inhibition of the catalytic activity of the proteasome *via* MG132 treatment showed a robust increase in APE2 protein abundance, with levels peaking at 16 h post treatment in U2OS cells (Fig. 1*A*). Similar observations of APE2 abundance (normalized to loading control tubulin) after MG132 treatment were also found in experiments conducted in human pancreatic cancer PANC1 (Fig. S1*A*) and human breast cancer MDA-MB-231 cells (Fig. S1*B*). Inhibition of translation by treatment with cycloheximide (CHX) resulted in the rapid decline of APE2 protein abundance with a half-life of ∼3.7 h (Fig. 1*B*). Inhibition of the proteasome prior to CHX treatment resulted in convincing recovery of APE2 protein abundance (Fig. 1*C*). APE2 abundance was calculated and normalized to loading control tubulin in these experiments (Fig. 1, *A–C*). These results demonstrate that APE2 is degraded by the proteasome in a cellular context.

After this initial validation that APE2 is degraded by the proteasome, we tested whether APE2 is modified by Ub. To demonstrate this modification, we generated a recombinant plasmid expressing Flag-tagged APE2 (designated as Flag-APE2) and transfected Flag-APE2 or control Flag into U2OS cells prior to proteasome inhibition and immunoprecipitation using anti-Flag antibodies (Flag-IP). When probing IP samples with anti-Ubiquitin antibodies, Flag-IP shows a ubiquitin-positive smear between 70kDa and 180kDa from cells transfected with Flag-APE2 but not Flag control, which is heavily enriched after MG132 treatment (Lane 3 and 4, Fig. 1*D*). Anti-APE2 antibodies not only recognized unmodified Flag-APE2 protein (∼70kDa) but also ubiquitinated Flag-APE2 in Flag-IP samples (∼180kDa) (Lane 3 and 4, Fig. 1*D*). These observations demonstrate biochemical evidence of APE2 ubiquitination, the first identified PTM of APE2. Taken together, our data strongly support that APE2 protein in cells is ubiquitinated and degraded by the UPS.

### APE2 K371 is critical for K48-linked poly-ubiquitination of APE2 and subsequent degradation

**Figure 2:**
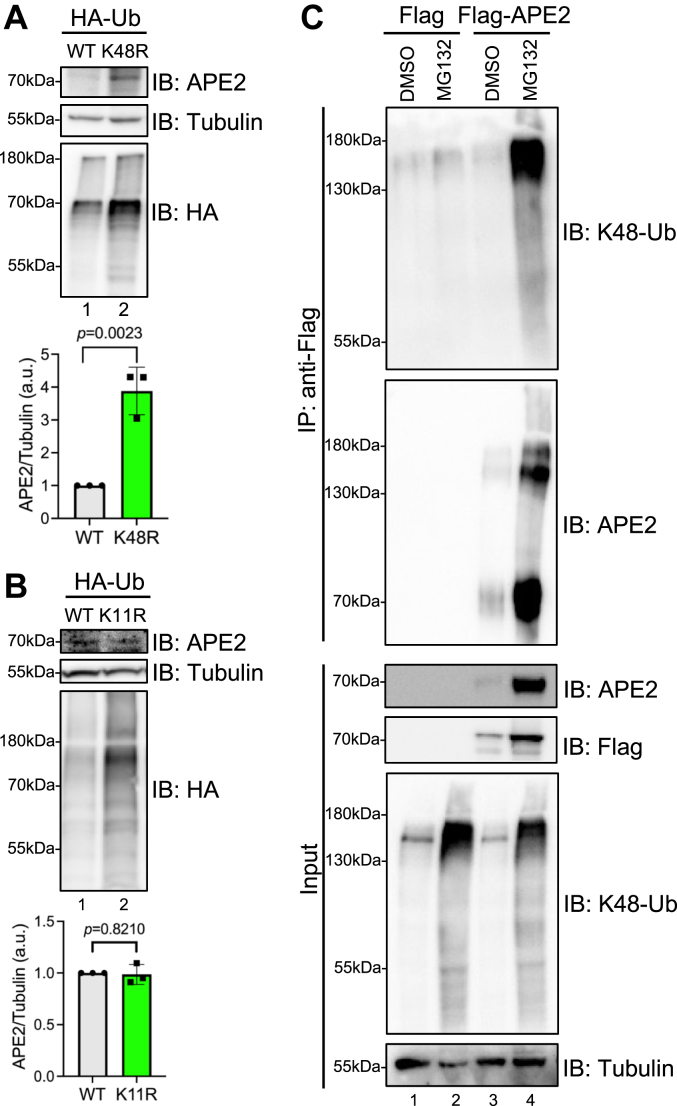
**APE2 is degraded by K48-linked polyubiquitin chains.***A*, transfection of HA-Ub-K48R results in a four-fold increase in APE2 protein abundance after 3 days expression in U2OS cells, compared with HA-Ub-WT. Unpaired two-tailed *t* test was performed on three biological replicates to generate indicated *p*-value. *B*, transfection of HA-Ub-K11R had no effects on APE2 protein abundance after 3 days expression in U2OS cells, compared with HA-Ub-WT. Unpaired two-tailed *t* test was performed on three biological replicates to generate indicated *p*-value. *C*, expression of Flag-APE2 but not Flag in U2OS cells and subsequent Flag-IP then immunoblotting with K48-Ub–specific antibody shows presence of K48-Ub–positive smear after MG132 treatment at similar molecular weight to chemical shift visualized on APE2 blot. APE2, AP endonuclease 2.

To further validate and characterize the key steps in APE2 protein degradation *via* the UPS, it was critical to establish the Ub linkage type responsible for targeting APE2 to the proteasome. Because the most common and best defined Ub linkage type associated with the UPS is K48-linked Ub, we attempted to identify this modification’s presence or absence. To validate that APE2 is degraded by K48-linked ubiquitination, we transfected recombinant WT HA-tagged Ubiquitin (HA-Ub-WT) or mutated HA-Ub at Lysine 48 to Arginine 48 (HA-Ub-K48R) (34, 35) to U2OS cells and found that after expression, HA-Ub-K48R caused significant upregulation of endogenous APE2 abundance compared with HA-Ub-WT (Fig. 2*A*). This observation is likely due to integration of HA-Ub-K48R into growing K48-Ub chains on APE2 and thus causing cessation of K48-Ub chain extension and increased APE2 abundance through proteasome evasion. Given that both K48 linkage and K11 linkage polyubiquitination might promote degradation (32, 36), we tested the potential role of K11-linked ubiquitination in APE2 degradation and found that transfection of HA-Ub-K11R expression plasmid to U2OS almost had no noticeable effects on APE2 protein abundance, compared with HA-Ub-WT (Fig. 2*B*). These observations suggest that APE2 protein is degraded mainly *via* the K48-linked polyubiquitination UPS in cells at least under unperturbed conditions.

Next, it was important to establish that APE2 is modified by K48-Ub chains. Because K48-Ub chains adopt a specific conformation, antibodies specific for this linkage type have been developed and well characterized (37). Use of anti-K48-Ub–specific antibodies after Flag-IP shows substantial K48-linked Ub-positive smear for Flag-APE2, especially after MG132 treatment (Fig. 2*C*). Importantly, a distinct chemical shift between 130kDa and 180 kDa can be observed on the APE2 blot from Flag-IP samples, a similar molecular weight to the major K48-Ub–positive smear (Lanes 3 & 4, Fig. 2*C*). However, anti-K11-Ub– and anti-K63-Ub–specific antibodies detected almost no appreciable signal from Flag-IP samples in a similar experiment design (Fig. S2, *A* and *B*). These observations indicate that APE2 protein is likely poly-ubiquitinated by K48-linked Ub chain which targets it to the proteasome for degradation.

**Figure 3:**
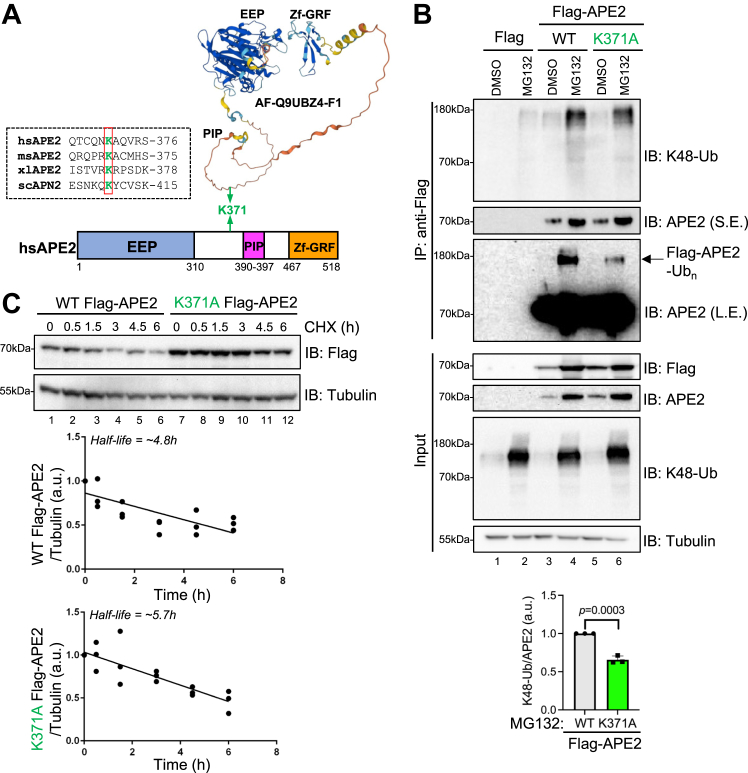
**APE2’s K371 is a major target for K48-Ub modification and proteasome-mediated degradation.***A*, a diagram of functional domains of human APE2, predicted structure of human APE2 protein by AlphaFold (AF-Q9UBZ4-F1), and amino acid alignment of peptides containing K371 within APE2 in humans (hsAPE2, GenBank no. NP_055296.2), mice (msAPE2, GenBank no. Q68G58.1), frog (xlAPE2, GenBank no. AAH77433.1), and budding yeast (scAPN2, GenBank no. NP_009534.1). *B*, Flag-APE2 WT, Flag-APE2 K371A, or Flag expression plasmid was transfected to U2OS cells with or without MG132 treatment. Input samples and Flag-IP samples were examined *via* IB analysis as indicated. Unpaired two-tailed *t* test was performed on three biological replicates to generate indicated *p*-value. *C*, WT and K371A-Flag-APE2 were transfected into U2OS cells prior to CHX pulse-chase experiments. APE2 abundance was normalized to tubulin from three biological replicates. Equation of line (WT Flag-APE2) Y=−0.07557∗X+0.8642 where Goodness of fit R square = 0.5983; (K371A Flag-APE2) Y=−0.09585∗X where Goodness of fit R square = 0.7084. APE2, AP endonuclease 2; EEP, Endonuclease/Exonuclease/Phosphodiesterase; L.E., long exposure; PIP, proliferating cell nuclear antigen interacting protein; S.E., short exposure; Zf-GRF, zinc finger motif containing GRF conserved residues.

According to PhosphoSitePlus analysis and the studies compiled therein, several amino acids in human APE2 such as K338, K371, K449, and K457 are good candidate residues for ubiquitination (17, 33, 38) (Figs. 3*A*, and S3*A*). An alignment of K371 in human APE2 shows conservation in mice, frogs, and yeast (Fig. 3*A*). Although the full length APE2 structure is not yet available, AlphaFold predicts that the K371 residue is located in an unstructured region of APE2 between the EEP domain and PIP domain (Fig. 3*A*). These observations prompted us to hypothesize that APE2 K371 may be ubiquitinated. To test this, we generated a mutant Flag-APE2 containing Lysine 371 mutation to Alanine (designated as Flag-APE2-K371A) and found that Flag-APE2-K371A showed a noticeable reduction in K48-specific Ub-positive smear signal from Flag-IP samples with the presence of MG132, compared with Flag-APE2-WT (lane 4 *versus* lane 6 and bottom quantifications/statistical analysis, Fig. 3*B*). Furthermore, the abundance of Flag-APE2-K371A was increased in total cell extracts compared with Flag-APE2-WT regardless of MG132 presence, suggesting that K371 is a major and critical, but may not singular, residue target for the proteostasis network responsible for APE2 degradation (Fig. 3*B*). To additionally validate K371 as a critical site of APE2 ubiquitination, high concentrations of Flag-APE2 immunoprecipitants were carefully immunoblotted to attempt to see an alteration in the chemical shit that appears at the same molecular weight as K48-Ub–positive smears. Long exposure of APE2 immunoblots revealed that K371A shows a distinct decrease in Ub-positive chemical shift providing direct evidence of APE2 modification by ubiquitin and successful reduction of the modified APE2 signal with K371A mutation (L.E. APE2 blot in IP samples, lane 4 *versus* lane 6, Fig. 3*B*). We observed a modest increase in the half-life of Flag-APE2-K371A as compared with Flag-APE2-WT (Fig. 3*C*). This observation is reminiscent of a similar chemical shift visualization strategy that has been previously utilized in proliferating cell nuclear antigen ubiquitination study (39).

As a complementary approach to further validate whether APE2 is ubiquitinated *via* K371, we transfect WT HA-tagged Ub (HA-Ub-WT) and a mutant HA-Ub in which all Lysine residues are mutated to Arginine excepting Lysine 48 (HA-Ub-K48only) (34, 35) and found that compared with HA-Ub-WT, HA-Ub-K48 only led to the reduction of Flag-APE2-WT with or without MG132 treatment (Fig. S3*B*). HA blot from Flag-IP samples after MG132 treatment showed a similar smear at about 180kDa (lane 2 *versus* lane 4, Fig. S3*B*), at the similar position as the Ub and Ub-K48 smears from Flag-IP samples (lane 4, Fig. 1*D*; lane 4, Fig. 3*B*). With HA-Ub-WT transfection, Flag-APE2-K371A led to an increase of APE2 abundance (lane 1–2 *versus* lane 5–6, Fig. S3*B*). Furthermore, HA blot from Flag-IP samples after MG132 treatment showed decreased ubiquitination in Flag-APE2-K371A by HA-Ub-K48 only, compared with Flag-APE2-WT (Lane 4 *versus* Lane 8, Fig. S3, *B* and *C*). These observations suggest that APE2 protein can experience K48-linked ubiquitination by both endogenous Ub protein and HA-Ub protein and provide an additional method showing that K371 is critical for APE2 K48-linked ubiquitination.

### MKRN3 acts as an E3 ligase upstream of APE2 ubiquitination and degradation in cells

In comparison to its paralog APE1 which is ubiquitinated by E3 ligase UBR3 (40), APE2’s E3 ubiquitin ligase responsible for its ubiquitination and regulation has remained unexplored. While the cell possesses only a handful of E1 and E2’s, there are upwards of 600 E3 ligases in human cells (41). Out of the several potential APE2-interacting proteins from EMBL-EBI IntAct molecular analysis, Makorin RING finger protein 3 (MKRN3), tripartite motif containing 37, and mitogen-activated protein kinase kinase kinase 1 are putative E3 ligases for APE2 ubiquitination (Fig. S4*A*). MKRN3 belongs to the RING Finger-type E3 ligase protein family and is involved in non-small cell lung cancer as a tumor suppressor; however, it remains unclear whether and how MKRN3 may interact and regulate APE2 ubiquitination and abundance (42, 43).

**Figure 4:**
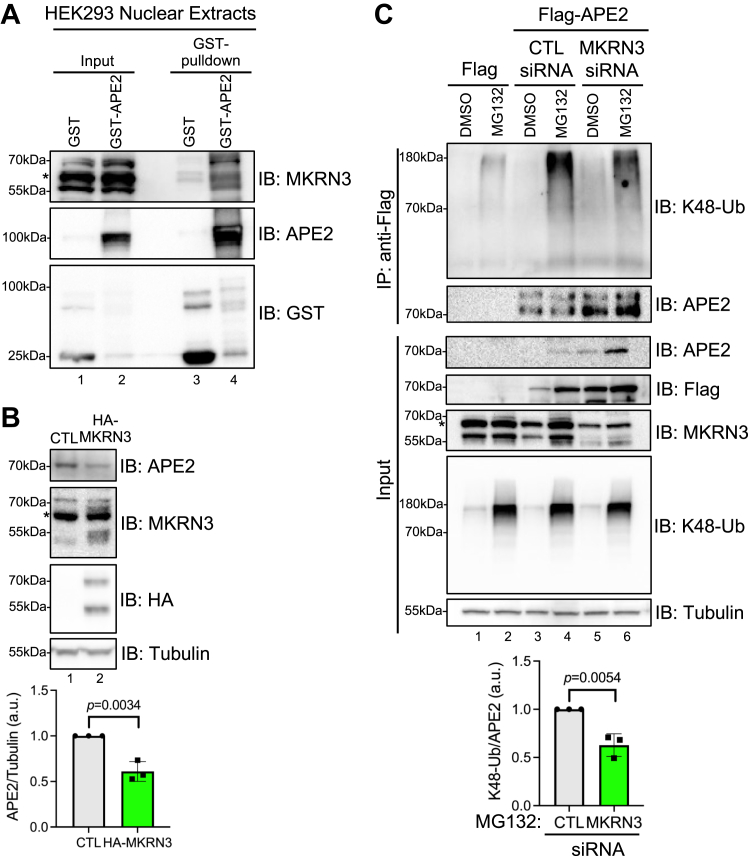
**MKRN3 acts as an E3 ligase upstream of APE2 degradation.***A*, GST-APE2 but not GST can pulldown MKRN3 from HEK293 nuclear extracts. *B*, HA-MKRN3 overexpression in U2OS cells shows a roughly 50% decrease in endogenous APE2 levels. Unpaired two-tailed *t* test was performed on three biological replicates to generate indicated *p*-value. *C*, Flag-APE2 IP after MG132 treatment with MKRN3 siRNA-mediated knockdown shows reduced K48-Ub–positive smears, compared to CTL siRNA-knockdown. Unpaired two-tailed *t* test was performed on three biological replicates to generate indicated *p*-value. ∗A possible nonspecific band overlapping with MKRN3 bands. APE2, AP endonuclease 2.

First, we wanted to test whether MKRN3 interacts with APE2. GST pull-down assays showed that GST-APE2 but not GST could pull-down endogenous MKRN3 protein from isolated nuclear extracts (Fig. 4*A*), suggesting that APE2 associates with MKRN3. If MKRN3 is the E3 ligase for APE2 ubiquitination, it is predicted that the abundance of APE2 would be reduced when MKRN3 is overexpressed. We found that the abundance of endogenous APE2 was indeed decreased about 50% when HA-tagged MKRN3 (HA-MKRN3) was overexpressed in U2OS cells (Fig. 4*B*). Furthermore, overexpression of HA-MKRN3 also led to reduced abundance of Flag-APE2 regardless of the MG132 pretreatment (lane 3–4 *versus* lane 5–6, Fig. S4*B*). Conversely, siRNA-mediated MKRN3-knockdown significantly decreased K48-Ub of Flag-APE2 and resulted in the upregulation of Flag-APE2 abundance under unperturbed and MG132 conditions (Fig. 4*C*). siRNA-mediated MKRN3-knockdown doubled the half-life of endogenous APE2 protein when subject to CHX pulse-chase experiment (Fig. S4*C*). Taken together, these results show that MKRN3 is critical for the stability of APE2 in human cells, likely through the catalyzation of K48-linked ubiquitination of APE2 and subsequent UPS-mediated degradation.

### Ubiquitination of recombinant APE2 protein was enhanced by the presence of MKRN3 protein in *in vitro* ubiquitination assays

**Figure 5:**
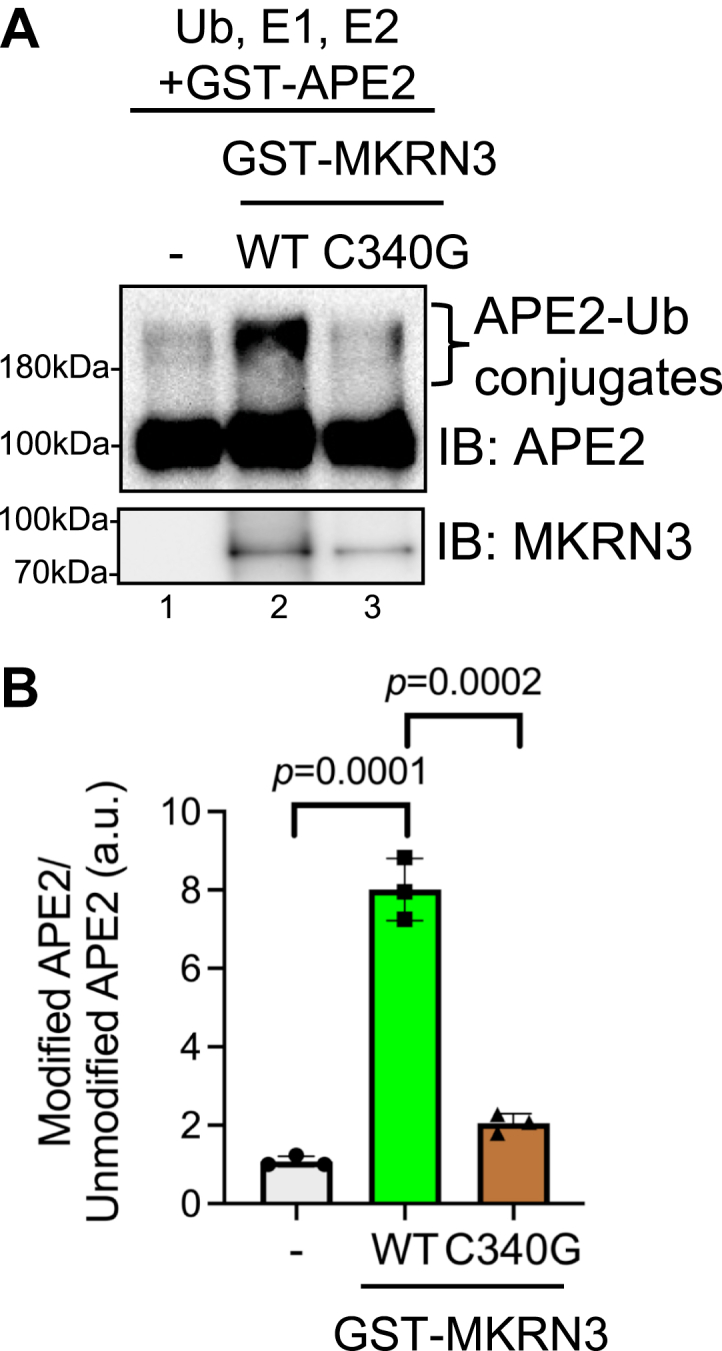
***In vitro* validation of APE2 as a substrate of MKRN3.***A*, GST-APE2 was incubated in the presence of UB, E1, and E2 with the absence or presence of WT GST-MKRN3 or catalytically dead C340G GST-MKRN3 (C340G) to show a ubiquitin positive smear (“APE2-Ub conjugates”) after incubation with WT-GST-MKRN3 but not with C340G-GST-MKRN3. A distinct band forms at the same APE2 molecular weight shift as *in vivo* experiments. *B*, quantification of Ub-modified APE2 normalized to unmodified APE2 was performed on three biological replicates followed by unpaired two-tailed *t* test to generate indicated *p*-value. APE2, AP endonuclease 2.

In order to directly visualize MRKN3-mediated APE2 ubiquitination, we utilized an *in vitro* ubiquitination assay containing recombinant Ub protein, recombinant ubiquitin activating enzyme E1 UBE1, and recombinant ubiquitin-conjugating enzyme E2 Ubc5a (44, 45). Previous study has identified and characterized that C340G mutation within MKRN3 led to its E3 ubiquitin ligase deficiency (45, 46), suggesting that C340G mutant MKRN3 can be utilized as a catalytically dead mutant for *in vitro* ubiquitination assays. Our results from the *in vitro* assay showed that GST-APE2 was efficiently ubiquitinated in the presence of purified WT-GST-MKRN3 protein but not significantly ubiquitinated in the presence of catalytically dead C340G-GST-MKRN3 protein or an E3-deficient setting (Fig. 5). These results further validate that APE2 is a direct substrate of MKRN3 E3 Ub ligase activity.

## Discussion

In this study, we have provided evidence of (i) APE2 protein abundance regulation *via* proteasome-mediated degradation (Fig. 1), (ii) K48-linked Ub modification of APE2 at K371 (Figs. 2 and 3), (iii) MKRN3’s upstream role as an E3 ligase for APE2 in cells (Fig. 4), and (iv) *in vitro* validation of MKRN3 E3 ligase activity on APE2 as a substrate (Fig. 5). These findings present and characterize the first PTM regulation of APE2, ubiquitination. Ubiquitination of APE2 *via* MKRN3 is, to the best of our knowledge, the first defined mode of cellular regulation of APE2 abundance. MKRN3 is critical for ubiquitination and degradation of poly-A–binding proteins such as PABPC1 for the prevention of Central Precocious Puberty and acts as a tumor suppressor gene through its E3 ligase activity (42, 43, 45, 47). Our study identifies APE2 as a new substrate of MKRN3 and presents new questions pertaining to APE2’s role in development, cell cycle regulation, and cellular transformation through its association with MKRN3. While questions still remain regarding the consequences of APE2 expression levels, here we present the first model for APE2 proteostasis which will inform future studies on APE2 protein abundance and its effects on ATR-Chk1 DDR signaling, cellular transformation, tumor suppressor activity, and clinical response to cisplatin chemotherapy.

Considering this correlation and the known functions of APE2, we can speculate that cells attempt to keep APE2 in a proverbial “Goldilocks” zone with two scenarios occurring with dysregulation: (1) low levels of APE2 results in unresolved complex 3′-termini, reduced MMEJ capacity, and failure to activate ATR-Chk1 DDR signaling from single-strand breaks, this in turn leads to genome instability which is a driver of transformation; and (2) high levels of APE2 increases single-strand DNA loads *via* its exonuclease activity which are vulnerable to DSB conversion and cause inappropriate ATR-Chk1 signaling, leading to genome instability which is a potential driver of transformation. Without sufficient understanding of how APE2 is regulated and the consequences of dysregulation, the clinical potential of APE2 is precluded (21). This leads to an important question: what is the optimal concentration range of APE2 protein in cells? The whole cell mass spectrometry analysis shows that human APE2 concentration is estimated to ∼23 nM in HEK293T cells (48). Better understanding of APE2 protein levels and its causes and consequences will enable researchers to further investigate in future whether APE2 is a viable target for chemotherapy, if APE2 is a driver or passenger of cancer development, and whether APE2 loss/overexpression is harmful or beneficial to genome stability in different cell types.

What are the potential implications of findings on APE2 ubiquitination and abundance in cancer development? From a query of 198,407 samples from 188,916 cancer patients in cBioPortal, 352 mutations including missense, truncating, and in-frame and splicing variants are found within APE2 gene. While APE2 A372T and A372V missense mutations were found from a non-melanoma skin cancer patient, an APE2 C368∗ nonsense mutation was found in a lung adenocarcinoma patient. Further investigations are needed to elucidate whether these variants near the K371 reside within APE2 from cancer patients may regulate APE2 interaction with its E3 ligase MKRN3 and/or APE2 abundance in genome integrity and cancer development.

Can APE2 be targeted for cancer therapeutics by modulating the basic mechanisms of APE2 ubiquitination and abundance? Previous studies have identified and characterized APE2 as a synthetic lethal target in homologous recombination-deficient cancers (1, 3). Downregulation of APE2 abundance *via* targeting MKRN3-mediated APE2 ubiquitination may be exploited in the future as a new approach for cancer treatment in homologous recombination-deficient cancer patients. Additionally, cisplatin-induced AKI and renal toxicity are the major side effect of cisplatin chemotherapy and likely due to cisplatin-mediated APE2 upregulation (21). A potential combinational therapy consisting of downregulating APE2 abundance (potentially *via* MRKN3-mediated APE2 ubiquitination and degradation) and cisplatin treatment could potentially offer new avenues for cancer therapeutics and decrease the cisplatin-dose–limiting effects of AKI caused by APE2 overexpression.

Overall, our study has demonstrated evidence of MRKN3-mediated APE2 ubiquitination and degradation *via* the UPS system. These basic mechanisms of APE2 ubiquitination and degradation provide new mechanistic understanding of APE2 abundance and its functions in genome integrity and cancer development and treatment in a cellular setting.

## Experimental procedures

### Cell culture, treatments, and cell lysate preparation

U2OS (HTB-96), PANC1 (CRL-1469), MDA-MB-231 (HTB-26), and HEK293 (CRL-1573) cells were purchased from ATCC and cultured in complete media (Dulbecco’s modified Eagle’s medium (Corning #MT10017CV), 10% Fetal Bovine Serum (R&D Systems #S11550), and 1% penicillin/streptomycin (Gibco #15140122). Cells were treated with MG132 (Selleckchem #S2619) dissolved in dimethyl sulfoxide at a working concentration of 500 nM for 24 h (unless specified) and CHX (Sigma #C6255) at a final concentration of 50 μg/ml dissolved in water. Negative control treatments for experiments including MG132 were performed with dimethyl sulfoxide carrier only.

For cell lysis, cells were washed with PBS (Gibco #10010023) and trypsin (Corning #25-053-Cl). The cells were collected by centrifugation and resuspended in ice-cold PBS followed by centrifugation. Cultured cells were lysed with lysis buffer (20 mM Tris–HCL pH 8.0, 150 mM NaCl, 2 mM EDTA, 0.5% NP-40, 0.5 mM Na_3_VO_4_, 5 mM NaF, 5 μg/ml of aprotinin, and 10 μg/ml of leupeptin). Lysates were centrifugated at 13,000 rpm for 30 min at 4 °C. The supernatants were transferred into fresh tubes for measuring protein concentrations *via* Bradford assays (Bio-Rad #5000006) and subsequent immunoblotting analysis.

### Preparation of recombinant plasmids and recombinant proteins

Recombinant plasmid Flag-APE2 was prepared by PCR coding sequences of *Homo sapiens APEX2* complementary DNA (GenBank Gene ID: 27301) with a pair of primers (FP#1 and RP#1, Table S1) and inserting into pN3-3x-Flag-Control vector through double digestion at EcoRI and Acc65I. GST-APE2 was generated in a similar manner to Flag-APE2 utilizing a pair of primers (FP#2 and RP#2, Table S1) before cloning into pGEX-4T1 vector through double digestions at Xho1 and EcoRI. To generate Flag-APE2-K371A plasmid, primers (FP#3 and RP#3, Table S1) were designed for primer-induced mutagenesis which was performed as previously defined (Table S1) (49). WT HA-MKRN3 plasmid was synthesized externally (VectorBuilder) using *MKRN3* cDNA (NCBI: NM_005664.4). Similarly, C340G HA-MKRN3 expression plasmid was synthesized with one nucleotide conversion T1078G in the coding region of *MKRN3* to ensure C340G mutation at the expected protein level. Primers were designed for WT or C340G MKRN3 cloning into a GST-expression vector pGEX-4T1 (FP#4 and RP#4, Table S1) using EcoR1 and Xho1 double digestion.

pN3-3x-Flag-Control was a gift from Guntram Suske (Addgene plasmid #107717; http://n2t.net/addgene:107717; RRID: Addgene_107717) (50). HA-Ubiquitin plasmid was a gift from Edward Yeh (Addgene plasmid #18712; http://n2t.net/addgene:18712; RRID:Addgene18712) (35). HA-Ubiquitin-K48R plasmid was a gift from Ted Dawson (Addgene plasmid # 17604; http://n2t.net/addgene:17604; RRID: Addgene_17604) (34). HA-Ubiquitin-K11R was a gift from Josef Kittler (Addgene plasmid # 121154; http://n2t.net/addgene:121154; RRID: Addgene_121154) (51). HA-Ubiquitin-K48only was a gift from Ted Dawson (Addgene plasmid #17605; http://n2t.net/addgene:17605; RRID: Addgene_17605) (34). All plasmids were validated by whole plasmid DNA sequencing (Plasmidsaurus). Recombinant GST, GST-APE2, WT, and C340G GST-MKRN3 were expressed in DE3/BL21 *Escherichia coli* cells after isopropyl 1-thio-β-d-galactopyranoside induction with the respective recombinant plasmid and purified using the vendor's procedures and as previously described (7, 8).

### Transfections and siRNA knockdown

U2OS cells were transfected using Lipofectamine 2000 (Thermo Fisher Scientific#11668019) using 1 μg of plasmid according to vendor procedures. For siRNA experiments, MKRN3 siRNA was purchased (Horizon Discovery #L-006581-00-0005) and used in combination with Lipofectamine RNAiMAX (Thermo Fisher Scientific#13778150) according to manufacturer protocols.

### Immunoprecipitation and GST pulldown

For immunoprecipitations, cells were lysed in EDTA-free RIPA buffer after 3 days of recombinant protein expression (50 mM Tris–HCl, 150 mM NaCl, 1% NP40, 0.5% sodium deoxycholate, 0.1% SDS, 5 μg/ml of aprotinin, and 10 μg/ml of leupeptin). Ten percentage of lysis was reserved and measured for protein concentration for downstream use in immunoblotting assays. The remaining lysis was incubated with 10 μl of Flag-beads (Sigma #M8823-5ML) before elution with Flag peptide (Thermo Fisher Scientific#A36805) according to manufacturer protocol. GST Pulldowns were performed as previously described (52, 53, 54).

### Immunoblotting Analysis and Antibodies

Immunoblotting of cell lysates was carried out as previously described (8, 52, 54). Antibodies used in this study are as follows: APE2 (as descripted previously (8, 55)), tubulin (Santa Cruz Biotechnology #sc-8035), Ub (Cell Signaling Technology #E412J), K48-Ub (Cell Signaling Technology #8081), K63-Ub(Cell Signaling Technology #5621S), K11-Ub (Sigma #SAB5701121), HA (Cell Signaling Technology #C29F4), Flag (Invitrogen: MA1-91878, Sigma #F1804), MKRN3 (Sigma Prestige Antibodies #HPA029494), GST (Santa Cruz: #sc-138).

### Quantification and statistical analysis

ImageJ was used to measure integrated density of proteins of interest. Background measurements were subtracted from this value prior to normalization of the protein of interest to tubulin (total cell extracts) or total protein (IP). Three biological replicates were quantified and then using GraphPad Prism software (version 10, www.graphpad.com), unpaired two-tailed t-tests were conducted. Linear regression and goodness of fit was quantified from three biological replicates using GraphPad prism software.

### In vitro ubiquitination assay

Recombinant human Ub (#U-100H-10M), recombinant human Ub-activating enzyme E1 (UBE1, #E-305-025), and recombinant human Ub-conjugating enzyme E2 (UbcH5a/UBE2D1, #E2-616-100) enzymes were purchased from R&D Systems. *In vitro* Ub assay was performed as previously described (45, 56).

## Data availability

All data described in the manuscript are present in the main text, figures, and the supplementary figures and tables.

## Supporting information

This article contains supporting information.

## Conflict of interest

The authors declare that they have no conflicts of interest with the contents of this article.
