# Supplementary material for: Ubiquitin-mediated regulation of APE2 protein abundance
Source: J Biol Chem. 2024 May 4;300(6):107337. doi: 10.1016/j.jbc.2024.107337 (PMC11157268; doi:10.1016/j.jbc.2024.107337)
Supplement: Supplemental Figures S1–S4 and Table S1 [file mmc1.pdf]

Supplementary Information

Ubiquitin-mediated regulation of APE2 protein abundance

Anne McMahon[1], Jianjun Zhao[2], Shan Yan [1,3,4] *

[1]Department of Biological Sciences, University of North Carolina at Charlotte, Charlotte, NC 28223, USA

[2]Department of Cancer Biology, Lerner Research Institute, Cleveland Clinic, Cleveland, OH 44195, USA

[3]School of Data Science, University of North Carolina at Charlotte, Charlotte, NC 28223, USA

[4]Center for Biomedical Engineering and Science, University of North Carolina at Charlotte, Charlotte, NC 28223, USA

*To whom correspondence should be addressed: Shan Yan

E-mail: shan.yan@charlotte.edu
ORCID: https://orcid.org/0000-0001-8106-6295

<u>List in SI:</u>

4 supplementary figures

1 Supplementary table

Supplementary figures and legends:

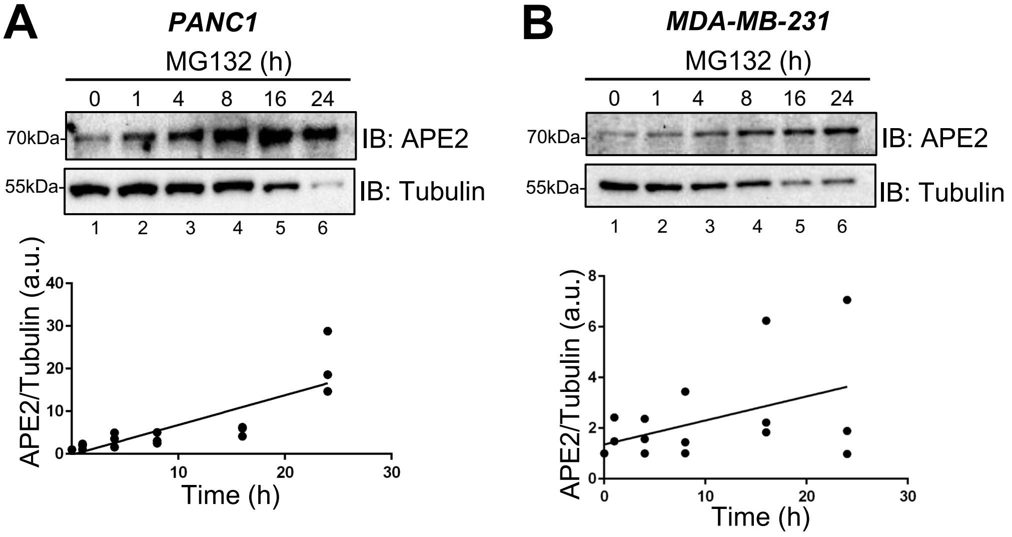

Figure S1. *A-B*, APE2 protein was immunoblotted, and protein levels were quantified from three biological replicates over a time course treatment of proteasome inhibitor MG132 in PANC1 (*A*) and MDA-MB-231(*B*) cells. Equation of line: (PANC1) Y = 0.6989*X - 0.1883 where Goodness of fit R square = 0.6974; (MDA-MB-231) Y = 0.09497*X + 1.355 where Goodness of fit R Square = 0.2295.

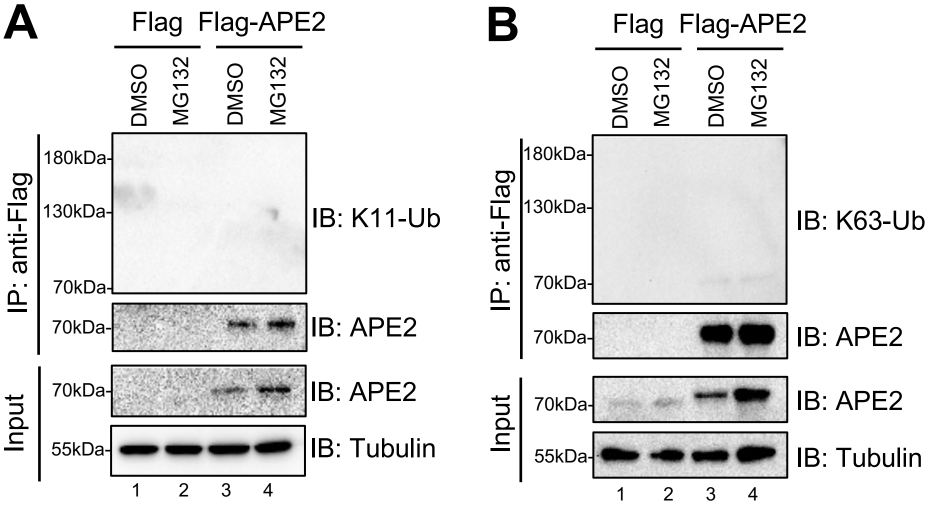

Figure S2. *A-B*, Flag-APE2 or Flag expression plasmid was transfected to U2OS cells prior to MG132 treatment and Flag-IP. Immunoblotting (IB) analysis with K11-Ub specific antibody (*A*) or K63-Ub specific antibody (*B*) from IP samples show no positive signal for these Ubiquitin linkage types present on Flag-APE2.

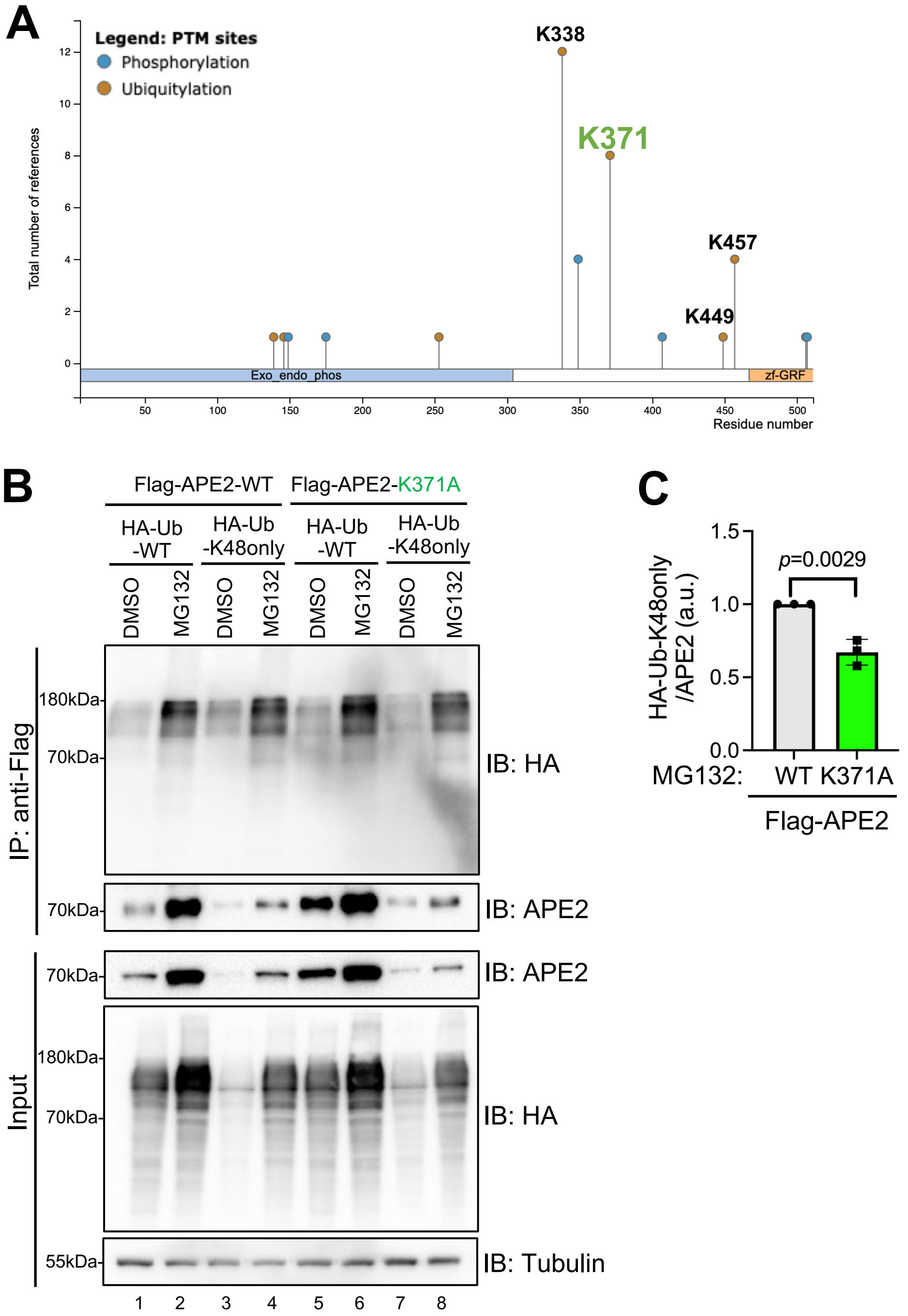

Figure S3. *A*, Schematic diagram of predicted lysine resides for ubiquitination and phosphorylation in human APE2 from PhosphoSitePlus analysis. *B-C*, Transfection of HA-Ub-WT or HA-Ub-K48only together with Flag-APE2-WT/K371 to U2OS cells. After MG132 treatment, Flag-IP samples and Input samples were examined via IB as indicated. Unpaired two-tailed t-test was performed on three biological replicates to generate indicated p-value.

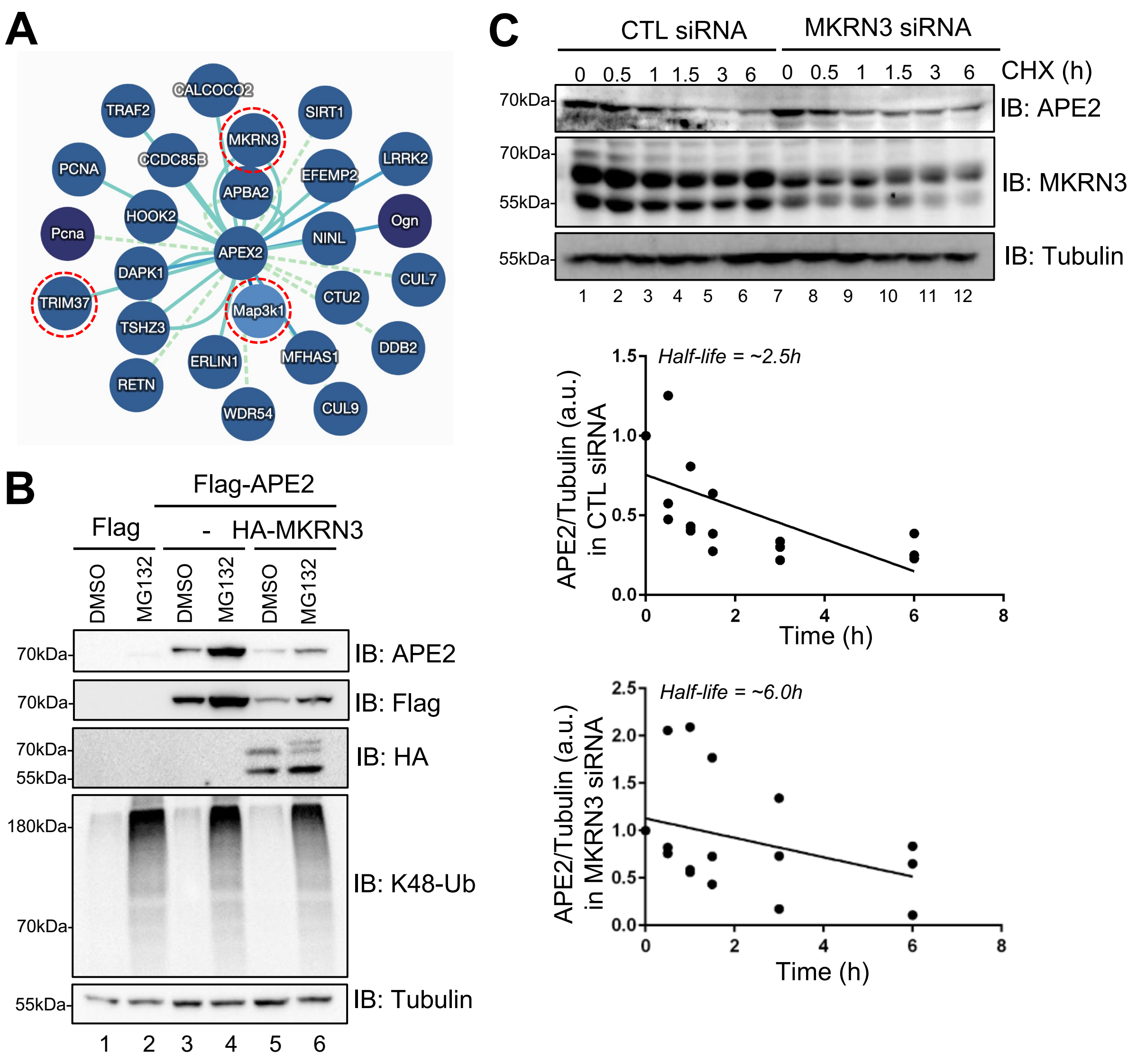

Figure S4. *A*, String analysis (EMBL-EBI IntAct) of predicted protein-protein interactions with APE2 where E3 ligases are marked by dashed red circles. *B*, Transfection of Flag-APE2 or Flag with HA-MKRN3 overexpression with or without MG132 treatment. Total cell lysates were examined via IB as indicated. *C*, Control (CTL) and MKRN3 targeting siRNA were transfected into cells prior to CHX pulse-chase experiments. APE2 abundance was normalized to tubulin from three biological replicates. Equation of line (CTL siRNA) Y=-0.1010*X+0.7559 where Goodness of fit R square = 0.4271; (MKRN3 siRNA) Y=-0.1025*X+1.130 where Goodness of fit R square = 0.1413.

Supplementary table:

Table S1. The sequence of oligos used in this study:

**FP#1:**	5'-GGGGGAATTCTGATGTTGCGCGTGGTGAGCTGG-3'
**RP#1:**	5'-GGGGGGTACCTCAGCTGGGCCTGCTCCAGAGGAAG-3'
**FP#2:**	5'-GGGGGAATTCATGTTGCGCGTGGTGAGCTGG-3'
**RP#2**	5'-GGGGCTCGAGTCAGCTGGGCCTGCTCCAGAGGAAG-3'
**FP#3**	5'-GGTACAGACATGCCAAAACGCAGCCCAAGTGCGCTCAACCAG-3'
**RP#3**	5'-CTGGTTGAGCGCACTTGGGCTGCGTTTTGGCATGTCTGTACC-3'
**FP#4**	5'-GGGGGAATTCATGGAAGAGCCTGCAGCTCCCT-3'
**RP#4**	5'-GGGGCTCGAGCTACAGAATCAAATTGAAATATTC-3'
